# The impact of patient self assessment of deformity on HRQL in adults with scoliosis

**DOI:** 10.1186/1748-7161-2-14

**Published:** 2007-10-15

**Authors:** Megan J Tones, Nathan D Moss

**Affiliations:** 1 Queensland University of Technology, Victoria Park Road, Kelvin Grove, 4059, QLD, Australia; 2 Queensland University of Technology, Beams Road, Carseldine, 4064, QLD, Australia

## Abstract

**Background:**

Body image and HRQL are significant issues for patients with scoliosis due to cosmetic deformity, physical and psychological symptoms, and treatment factors. A selective review of scoliosis literature revealed that self report measures of body image and HRQL share unreliable correlations with radiographic measures and clinician recommendations for surgery. However, current body image and HRQL measures do not indicate which aspects of scoliosis deformity are the most distressing for patients. The WRVAS is an instrument designed to evaluate patient self assessment of deformity, and may show some promise in identifying aspects of deformity most troubling to patients. Previous research on adolescents with scoliosis supports the use of the WRVAS as a clinical tool, as the instrument shares strong correlations with radiographic measures and quality of life instruments. There has been limited use of this instrument on adult populations.

**Methods:**

The WRVAS and the SF-36v2, a HRQL measure, were administered to 71 adults with scoliosis, along with a form to report age and gender. Preliminary validation analyses were performed on the WRVAS (floor and ceiling effects, internal consistency and collinearity, correlations with the SF-36v2, and multiple regression with the WRVAS total score as the predictor, and SF-36v2 scores as outcomes).

**Results:**

The psychometric properties of the WRVAS were acceptable. Older participants perceived their deformities as more severe than younger participants. More severe deformities were associated with lower scores on the Physical Component Summary Score of the SF-36v2. Total WRVAS score also predicted Physical Component Summary scores.

**Conclusion:**

The results of the current study indicate that the WRVAS is a reliable tool to use with adult patients, and that patient self assessment of deformity shared a relationship with physical rather than psychological aspects of HRQL. The current and previous studies concur that revision of the WRVAS is necessary to more accurately represent the diversity of scoliosis deformities. Ability to identify disturbing aspects of deformity could potentially be improved by evaluating each WRVAS items against indicators of pain, physical/psychosocial function, and self image from previous measures such as the SRS, SF-36 or BSSQ-deformity.

## Background

Outcomes from psychosocial and health related quality of life (HRQL) studies indicate that body image is a complex and significant issue for patients with scoliosis and their clinicians [1,2]. According to the body image literature, medical conditions threaten the stability of patient body image via changes to bodily sensation, functioning and appearance [3]. In particular, disfigurement or deformity can promote a negative self image within the individual, who may also experience difficulties with social interaction due to potential adverse reactions from others as a result of the visibility of their condition [4]. As scoliosis is rarely life threatening, the clinician's decision to perform scoliosis surgery on adolescents hinges on current and prospective spinal deformity, with patient HRQL and surgical considerations performing auxiliary roles in the decision making process [5]. Cosmetic issues and physical symptoms are the key indicators for scoliosis surgery in adult patients, even though further curvature progression is extremely unlikely [1]. However, clinical assessments of scoliosis correlate poorly with patient self perceptions of deformity and self reported HRQL [5,6]. Assessment of body image and factors likely to influence body image (HRQL) is important for scoliosis patients, as difficulties in these areas may have an adverse affect on treatment compliance and satisfaction in adolescence, and limit psychosocial functioning in adult life [7-9].

Recent attempts to map radiographic and treatment variables to HRQL and body image outcomes in the literature are summarized in Table 1 and Table 2. Earlier HRQL and psychosocial studies are summarized elsewhere [2]. Few consistencies emerge in the literature, and factors such as age, gender and psychological health confound the relationship between clinical measures of scoliosis deformity and body image or HRQL outcomes [7,10,11]. It can be seen that for adolescent patients treated conservatively, brace wear exerts a greater impact on body image and HRQL than the deformity itself [12]. Correlations between radiographic measures and self reported outcome measures range from mild to moderate amongst adolescent surgical candidates, with a tendency for stronger correlations in the Self Image, Function and Pain domains HRQL, and poorer outcomes amongst patients with thoracic curvatures [13-16]. With the exception of saggital balance, radiographic measures were shown to be even poorer indicators of body image and HRQL outcomes in adults [10,17,18]. The nature of scoliosis deformity is subject to age graded changes, and age itself is associated with poorer HRQL regardless of disease status. Psychosocial studies of adult scoliosis patients have also revealed limitation in social and intimate relationships due to physical difficulties in participation, fear of injury or self consciousness [9,17,19,20].

**Table 1:** Studies Evaluating Radiographic/Topographic and Treatment Indicators against Body Image and HRQL Outcomes in Adults with Scoliosis

**Citation**	**Participants**	**R/T/Indicator**	**BI/HRQL Outcome**	**Findings**
**Adult Studies**				

Bridwell et al [41]	56 adult patients (50 female, age range 21–60+). All prior surgical treatment. Mean Cobb angle 59.5° preoperative, 29° 2 yr postoperative	Curvature pattern, curve magnitude, treatment factors	SRS-22, SF-12, Oswestry Disability Index (ODI)	*Curvature pattern: *Similar rate of improvement in HRQL following surgery regardless of age/curvature pattern. *Treatment (surgery to reduce curve magnitude): *pre op. to 1 year post op. = improvements in all HRQL domains except for SF-12 Mental Component Summary (MCS) and SRS-22 Function; pre op. to 2 year post op. = improvements in all HRQL domains except for SF-12 MCS; 1 year post op. to 2 year post op. = improvement in SRS-22 Pain. The most significant improvements occurred for SRS-22 (Self Image, Total score, Pain), ODI, SF-12 Physical Component Summary (PCS), SRS Mental, and SF-12 Mental Component Summary. Older age = poorer outcomes on SF-12 PCS
Glassman et al [42]	161 matched pairs of surgically/non-surgically treated adult patients (286 female, age range 18–80). Mean Cobb angle 43° non-surgical group, 53° surgical group	Treatment type	SRS-22, SF-12, Oswestry Disability Index (ODI)	*HRQL/Other symptoms: *Non surgically treated group had a higher incidence of surgical risk factors (heart disease, overweight). General Health as measured by SF-12 was poorer in non surgical group. Surgical patients had a higher incidence of back/leg pain, and lower scores on the Role Physical and Bodily Pain domains of the SF-12. S urgical patients were more likely to report that the shape of their back had changed over the last 10 years and that they were very unhappy with the shape of their back. They also rated the appearance of their trunk as fair (compared to good amongst non surgical patients), and were more likely to state that their back limited personal relationships. Deciding factors for not selecting surgery were: older age, higher Body Mass Index Deciding factors leading to surgery were: lower SRS Self Image scores, larger thoracic curvature, greater back pain (ODI)
Glassman et al [10]	298 adult patients (84% female, age range 18–87). 126 prior surgical treatment. Curvature >30° or significant spinal deformity.	Curvature pattern, curve magnitude, coronal and sagittal balance	SRS-22, SF-12, Oswestry Disability Index (ODI)	*Curvature pattern: *Thoracic curvature associated with lower pain and better functioning for all patients, and better self image for surgically treated patients *Coronal balance: *Coronal shift greater than 4 cm associated with poorer functioning and greater pain in non surgically treated patients. *Sagittal balance*: Positive sagittal balance associated with greater pain lower function and poorer self image and social functioning.
Weinstein et al [17]	117 patients (89% female, age range 54–80 years). No surgery. Mean Cobb angle 85–90° in thoracic/thoracolumbar, 49° in lumbar curvatures.	Cobb angle, degree of apical rotation	Adapted Body Satisfaction Scale	Body satisfaction shared a low correlation with radiographic measures (*r *= -0.08 to -0.32). Patients reported difficulties purchasing clothes, lower physical capacity and self consciousness.
Schwab et al [18]	95 patients with AIS or degenerative scoliosis (62 female, mean age 59 years). No prior surgery. Cobb angle >15°	Radiographic indicators, pain	Visual analogue scale (VAS)	Mean Cobb angle was 28° (thoracic) and 38° (thoracolumbar/lumbar). Moderate pain reported (58 out of 100 on VAS). *Radiographic indicators: *Lumbar lordosis, thoracolumbar kyphosis, L3 Endplate angle, L4 Endplate angle and Olisthy associated with pain (VAS)

**Table 2:** Studies Evaluating Radiographic/Topographic and Treatment Indicators against Body Image and HRQL Outcomes in Adolecents with Scoliosis

**Adolescent Studies**				
Donaldson et al [5]	40 patients (32 female, mean age 14.5 years). 14 surgically treated. Mean Cobb angle for surgical candidates 63.9°, non surgical candidates 37.4°	Described in findings	3 questions: shame connected to body and appearance in swimsuit, poor shape	*Radiographic indicators: *Patients were classified according to severity of deformity as evaluated by radiographic indicators of curvature pattern/magnitude, coronal and sagittal measures and angle of trunk inclination. There was no correlation between patient body image and surgical recommendation. Patients not recommended for surgery had a slightly poorer body image than patients recommended for surgery.
Weiss et al [36]	63 patients (59 girls, mean age 13.6 years). Conservatively treated. Mean Cobb angle 43.7°	Treatment type	BSSQ – Brace	*Treatment type: *Distress associated with brace wear was reduced in the Cheneau light brace compared to previous bulkier brace models.
Kotzicki et al [12]	111 girls (mean age 14.2 years). 51 treated via exercises, 10 awaiting surgery, 50 treated by brace. Mean Cobb angle 42.8°	Curvature magnitude, angle of trunk inclination, treatment type	BSSQ – Brace, BSSQ – Deformity	*Curvature magnitude: *negative correlation between Cobb angle (*r *= -.34) and BSSQ-D *Angle of trunk inclination: *negative correlation between Bunnell primary curve rotation (*r *= -.34) and Bunnell sum of rotation (*r *= -.33) and BSSQ-D *Treatment type: *Conservatively treated patients experienced lower stress associated with their deformity (BSSQ-D = 17–18) than presurgical patients (BSSQ-D = 12). Patients treated via brace experienced greater stress associated with brace wear (BSSQ-B = 9) than deformity (BSSQ-D = 18).
Botens- Helmus et al [37]	62 patients (55 girls, mean age 14.5 years). Treated via brace. Mean Cobb angle 40°	Treatment	BSSQ – Brace	*Treatment: *23% of patients reported strong stress associated with brace wear (BSSQ-B: 0–8), 50% reported medium stress (BSSQ-B: 9–16), and 23% reported low stress (BSSQ-B: 17–24). The most stressful part of brace wear was concealment of the brace with clothing and hair style, while the least stressful element was avoidance of activities and hobbies due to brace wear.
Weiss et al [13]	206 patients (gender unspecified, mean age was 15.7 years. Treatment not specified. Mean Cobb angle 35.8°	Curvature magnitude/pattern	BSSQ – Deformity	*Curve magnitude/pattern: *Patients meeting plausibility criteria reported low stress associated with deformity (BSSQ-D = 19.97), while patients who did not meet plausibility criteria reported medium stress (BSSQ-D = 15.9). Negative correlation between Cobb angle and BSSQ-D (*r *= -0.19 plausible met; *r *= -0.54 plausible not met). Negative correlations between Cobb angle and BSSQ-D scores were strongest for thoracic curvatures (*r *= -0.49), and weakest for lumbar curvatures (*r *= -0.27)
Vasiliadis et al [38]	36 patients (32 female, mean age 13.9 years). Treated via brace. Mean Cobb angle 28.2°	Curvature pattern, magnitude and angle of trunk inclination	BraceQ	Areas of HRQL most affected in patients were physical functioning and vitality, followed by emotional functioning and self esteem/aesthetics. *Curvature pattern/magnitude: *negative correlation between lumbar Cobb angle and school activity scale (*r *= -0.72) *Curvature pattern/angle of trunk inclination: *negative correlation between social functioning scale and both lumbar (*r *= -0.67) and thoracolumbar (*r *= -0.66) ATI
Vasiliadis et al [39]	28 children (25 female, aged 9 to 18 years). Treated via brace. Mean Cobb angle approx 25°	Curvature magnitude, change in magnitude	BraceQ	*Curvature magnitude: *Mild scoliosis (curvature less than 30 degrees) was associated with better scores on the BrQ than moderate scoliosis (curvature of 30 to 38 degrees). Reduction in curvature magnitude over time was associated with increases in BrQ scores, while deterioration of curvature was associated with decreases in BrQ scores.
Smith et al [40]	128 patients (111 female, mean age 16.4 years). Surgically treated. Mean Cobb angle 56.1° preoperative, 33° postoperative	Curvature pattern/magnitude, rib prominence, other radiographic measures	Appearance of shoulder blades, shoulders, waist and body image (QLPSD)	*Curvature pattern/magnitude: *Magnitude of King type 1 curvature (thoracic) and King Type 3 curvature correlated with appearance of shoulder blades (1: *r *= 0.56; 3: *r *= 0.49). *Frontal spinal balance: *correlated with waist appearance (*r *= 0.25) *Shift from apex to central sacral line: *Type 1 (lumbar) correlated with waist (*r *= 0.68) and body image (*r *= 0.67); Type 2 (lumbar) curvatures correlated with appearance of shoulders (*r *= 0.39), waist (*r *= 0.40) and body image (*r *= 0.34); Type 3 correlated with shoulder blade appearance (*r *= 0.55) *Pelvic balance: *correlated with waist appearance (*r *= 0.31) and body image (*r *= 0.32)
Climent et al [14]	175 patients with AIS (152 female, mean age 18.9 years). 85 treated via surgery, 45 orthosis, 45 observation. Mean Cobb angle 28°	Curvature pattern, curve magnitude, treatment type	SRS-22	*Curve pattern: *higher pain for patients with single curvatures *Curve magnitude*: negatively correlated with Pain (*r *= -0.41), Function (*r *= -0.29), Self Image (*r *= -0.28), Mental Health (*r *= -0.33), Satisfaction (*r *= -0.30) and Total RS Scores (*r *= -0.43) *Treatment type: *surgically treated patients had a better self image and were more satisfied with treatment than patients treated via orthosis (attributable to reduction in curve magnitude)
Asher et al [15]	61 patients with AIS (50 female, mean age 15.5 years) Preoperative. Mean Cobb angle 63°	Curvature pattern, curve magnitude, radiographic and typographic indicators	SRS-22	*Curvature pattern: *Suzuki Hump Indices 3 and 5 differed according to curvature pattern, although there were no differences in SRS Outcomes for curvature pattern. King Mode Type III and IV are strongly correlated with Function (*r *= -0.53), Self Image (*r *= -0.46) and Total SRS scores (*r *= -0.45) *Curvature magnitude: *negatively correlated with Function (*r *= -0.39) *Typographic indicators: *negative correlation between Suzuki Hump Index 1 (rib hump at scapula level) and Function (*r *= -0.45), Self Image (*r *= -0.36) and Total SRS score (*r *= -0.37) There was no relationship between waist crease asymmetry, angle of trunk inclination, or posterior trunk symmetry index measures and SRS-22 outcomes.
Asher et al [16]	168 patients with AIS (145 female, mean age of 14 years). Not surgically treated. Mean Cobb angle of 30° (braced/observation) and 61° (surgical candidates).	Curvature pattern, curve magnitude, angle of trunk inclination (ATI), treatment type	SRS-22	*Curve pattern: *no relationship between curve pattern and SRS outcomes *Curve magnitude: *negatively correlated with Pain (*r *= -0.37), Self Image (*r *= -0.50), Function (*r *= -0.27), Mental Health (*r *= -0.27) and Total SRS score (*r *= -0.48). *ATI: *negatively correlated with Pain (*r *= -0.30), Self Image (*r *= -0.47), and Total SRS score (*r *= -0.39). *Treatment factors: *Patients with curvatures exceeding 40 degrees had poorer self image if surgery was recommended, compared to current conservative management.
Wilson et al [11]	265 patients with AIS (86% female, mean age 14.7 years). Surgically treated. Mean Cobb angle 52.5° preoperative, postoperative not stated	Curvature pattern/magnitude, coronal and sagittal indicators of deformity	SRS-24	*Curve pattern/magnitude: *Thoracic curvature magnitude and Pain (*r *= -0.22), General Self Image (*r *= -0.23) and Function (*r *= -0.18) and Total SRS (*r *= -0.22). Upper thoracic curvature and Function (*r *= -0.19). Lumbar curve and Pain (*r *= -0.20), General Self Image (*r *= -0.23) and Total SRS (*r *= -0.26). *Coronal-axial deformity: *Correlates with Pain (*r *= 0.24), General Self Image (*r *= 0.24), Function (*r *= 0.17) and Total SRS (*r *= 0.26). *Sagittal deformity: *No correlation.
Koch et al [7]	42 adolescents (32 female); mean age, 14.5 yr (range, 12–18 yr); postsurgical	Curvature pattern and magnitude Satisfaction with treatment	OFFER Self-image Q Revised; Multidimensional Body Self Relations Questionnaire postsurgery questionnaire	*Curvature pattern: *Patients with King Type II/III curvatures also had a lower BMI and were lower in menarchal status. They were more likely to report neutrality or dissastifaction with surgical outcomes. *Satisfaction with surgery: *Neutrality/dissatisfaction with surgery was related to lower scores on the OFFER Self Image Q prior to surgery, and lower scores on the Body Self Relations Q post surgery (especially low satifaction with mid/upper torso).

Assessment of body image in scoliosis patients has been limited to written questions about perceptions of attractiveness in clothing or bathing suits, satisfaction with the body or back, and psychosocial distress as a result of deformity or brace wear [17,19,21,22]. However, few attempts [5,17] have been made to qualify which aspects of deformity are the most distressing for patients. Such information would be useful for clinical decisions, such as whether or not to perform thoracoplasty in addition to spinal fusion, or whether to recommend conservatively treated patients for surgery. The most concentrated effort in this area was the development of a visual analogue scale to quantify patient self assessment of deformity by Sanders *et al *[23].

Known as the Walter Reed Visual Assessment Scale, the scale features seven items which address visual aspects of scoliosis including: body curve, head pelvis, rib prominence, shoulder level, flank prominence, scapula rotation and head rib pelvis (*cf *[23-25]). Each item consists of five illustrations scaled to indicate worsening deformity via higher scores. The WRVAS is not a body image scale as such. Rather, it is intended to assess the patient's perception of their deformity without cognitive or emotional connotations [23]. Table 3 summarizes the findings of previous studies using the WRVAS. Subsequent studies following initial development have demonstrated stronger and more consistent correlations between the WRVAS and HRQL outcomes than the radiographic studies outlined in Tables 1 and 2. This suggests that the WRVAS is a more accurate reflection of the impact of scoliosis deformity on patient body image and HRQL than radiographic indicators. As with the HRQL measures described in Tables 1 and 2, an attempt to map WRVAS outcomes to radiographic indicators yielded inconsistent findings. Assessment of patient perception of scoliosis deformity provides information unique to radiographic data, and due to its clinical relevance further investigation is warranted.

**Table 3:** Previous studies utilizing the WRVAS

Citation	Participants	Instruments/Variables	Findings	
Petruskevicius, Laursen & Lemche, *et al *[34]	26 patients who had undergone surgery (pedicle screw instrumentation)	WRVAS SRS (version undefined) Radiographic measures (undefined). WRVAS administered pre and post operatively.	Authors stated that : The WRVAS demonstrated a "good correlation" with some domains of the SRS, especially Self Image. The WRVAS "correlated well" with reductions in curve magnitude as a result of surgery.	
Sanders, Polly & Cats-Baril, *et al *[23]	182 patients (mean age 14.7 years, 82% female). 133 parents (of patients).	WRVAS Type of treatment undertaken/recommended (groups: not scoliosis, observation, brace treatment, surgery recommended) Curve magnitude	**Reliability: **Inter rater reliability between parents and patients was acceptable (Spearman's rho = 0.8). Individual item correlations (Spearman's rho) range from 0.4 to 0.74 for patients, and 0.36 to 0.76 for parents (*p *< 0.05).	**Validity: **Significant correlation between curve magnitude and total WRVAS score (*p *< 0.01), differentiates between curves greater or less than 30 degrees. Scores showed clear distinctions between treatment type/recommendation, with scores increasing from "not scoliosis" through to "surgery recommended" (*p *= .04).
Bago, Climent & Pineda, *et al *[35]	32 patients (mean age 17.9 years, range 13–40, 5 male).	WRVAS SRS-22 Curve magnitude	**Reliability: **Internal consistency (Cronbach's alpha) = 0.88. No evidence of collinearity.	**Validity: **Medium to strong correlations between all items of the WRVAS and Cobb angle (range *r *= 0.04 to *r *= 0.77). Strong correlation between total WRVAS score and total SRS-22 (-.63, *p *= .0001).
Pineda, Bago & Gilperez, *et al *[24]	70 patients (mean age 19.4 years, range 12–40, 10 male).	WRVAS SRS-22 Curve magnitude	**Reliability: **Internal consistency (Cronbach's alpha) = 0.90 (same for under 18 and over 18 groups). No evidence of collinearity.	**Validity: **Medium to strong correlations of WRVAS items with Cobbmax (range *r *= .41 to .71, *p *< .01). Strong correlation between total WRVAS score and Cobbmax (*r *= .69, *p *< .0001). Medium to strong correlations between SRS-22 domains and total WRVAS score (range *r *= -.40 to -.57, *p *= .0001). Strong correlation between total WRVAS score and total SRS-22 (*r *= -.54, *p *= .0001). Results identical for both age groups.
Bago, Climent, Pineda *et al *[25]	101 patients (mean age 19.4 years, range 10–40, 15 males).	WRVAS Curvature pattern (groups: (30 thoracic, 39 double major, and 32 thoracolumbar) Radiographic measures		**Validity: **Curvature magnitude (proximal thoracic, main thoracic, thoracolumbar and lumbar), main thoracic apical vertebra rotation and apical vertebra offset and *Cobbmax *shared a significant correlation with total WRVAS score and all WRVAS items (with the exception of main thoracic apical vertebra rotation. The radiographic measures: T1 offset from central sacral line, difference in shoulder level and thoracolumbar apical vertebra offset and rotation were unrelated to WRVAS outcomes. The WRVAS was not able to discriminate between different curvature types, although scores were differentiated according to curvature magnitude. The WRVAS demonstrated good correlations with equivalent radiographic measures on the items Body Curve (1), Rib Prominence (2), Head Rib Pelvis (4) and Scapular Rotation (7), but not Flank Prominence (3), Head Pelvis (5) or Shoulder Level (6).

One notable omission in studies utilizing the WRVAS is the evaluation of WRVAS scores against the Short Form Health Survey (SF-36), version 2. The current study will involve the administration of the WRVAS and Short Form Health Survey, version 2 [SF-36v2] to a sample of adult scoliosis patients in order to determine the impact of patient self assessment of deformity on a HRQL instrument widely used in and populations, and further validate the WRVAS.

## Methods

Cross sectional methodology was used to determine the reliability and construct validity of the WRVAS. Support groups, orthopaedic specialists and a large metropolitan university were approached in 2004 and 2005 in an attempt to obtain equal samples of males and females over 18 years of age with scoliosis. The total sample included 13 males and 63 females. Participants completed a questionnaire package which included a form to report age and gender, the Short Form Health Survey, Version 2 (SF-36v2), and the WRVAS.

### SF-36v2 questionnaire

The SF-36v2 is an updated version of the SF-36. Currently, the SF-36 is the most popular health related quality of life (HRQL) instrument used on adult populations with scoliosis [26,27]. The second version has been updated to simplify the layout, wording and response formats to minimize cultural bias [28,29]. Like its predecessor, the SF-36v2 is composed of eight subscales Physical Functioning (PF), Role Emotional (RE), Role Physical (RP), Bodily Pain (BP), Social Functioning (SF), Mental Health (MH), Vitality (VT) and General Health (GH). These subscales can be summarized into Physical Component Summary (PCS) and Mental Component Summary (MCS) scores. All scales are reported as T scores, which correspond to a mean of 50 [29].

### WRVAS questionnaire

As described earlier, the WRVAS is a seven item scale designed to evaluate patient perception of spinal deformity. Scores are obtained by totaling responses to each of the seven questions [24]. For each item the minimum possible score is 1 and the maximum is 5. The lowest possible score for the total is 7, while the highest possible total score is 35.

### Statistical Analysis

Previous validation studies of the WRVAS set precedence for statistical report [24-26]. As such, the means and standard deviations for each WRVAS item and the total WRVAS score, along with floor and ceiling effects were reported.

Internal consistency was evaluated via Cronbach's alpha to assure that all items measured a common underlying construct. According to Nunnally [30] a Cronbach's α of 0.7 is considered acceptable, while a value of 0.8 is 'good', and a value of 0.9 is 'excellent.'

Collinearity statistics in the form of tolerance and the variance inflation factor were also examined to identify multicolinearity amongst items in the WRVAS. Tolerance values greater than 0.1 and less than 10 indicate that all items of the WRVAS are unique in their contribution to the measurement of scoliosis deformity, in that none of the items are strongly intercorrelated and redundant [24,31].

Construct validity was addressed by correlating each item of the WRVAS and total WRVAS score with each scale of the SF-36v2. Two multiple regression analyses were also conducted to determine the capacity of the WRVAS to predict Physical and Mental Component Summary scores of the SF-36v2. Specifically, total WRVAS score served as the independent variable in each analysis, whilst Physical and Mental Component Summary acted as dependent variables. Age and gender were also examined in the correlations and multiple regressions for the purposes of statistical control.

## Results

Five female participants were excluded from the sample due to insufficient questionnaire completion. This resulted in a final sample of 13 males and 58 females or a ratio of 1:4.5, which is a similar ratio to another published study [27], and reflects the clinical population of scoliosis patients [32]. Missing data was substituted for participant subscale means in 13 cases (5 male), where at least four items of the WRVAS or half of the items of a subscale within the SF-36v2 was completed. The mean (standard deviation) participant age was 33 (12.7) years, and age ranged from 17 to 66 years.

The mean (standard deviation) of each WRVAS item and total WRVAS score is reported in Table 4. The percentage of participants who scored the maximum and minimum scores on each scale are also presented in Table 4. Total WRVAS scores were comparable for males and females. Statistically significant correlations were found between Age and Body Curve (*r *= .327), Head Pelvis (*r *= .255), Rib Prominence (*r *= .309), Flank Prominence (*r *= .351), Scapula Rotation (*r *= .297), Head Rib Pelvis (*r *= .342), and total WRVAS score (*r *= .339). This indicated that older participants assessed their deformity to be more severe than younger participants.

**Table 4:** Means, standard deviations, minimum and maximums of the WRVAS

Item	Mean	Stand. Dev.	% Minimum	% Maximum
Body Curve	2.55	1.16	19.7%	9.9%
Head Pelvis	2.34	1.12	25.4%	4.2%
Rib Prominence	1.99	0.96	33.8%	1.4%
Shoulder Level	2.38	1.05	18.3%	4.2%
Flank Prominence	2.25	1.01	22.5%	4.2%
Scapula Rotation	2.40	1.06	19.7%	5.6%
Head Rib Pelvis	2.21	1.03	25.4%	4.2%
WRVAS Total	16.12	6.14	2.8%	1.4%

### Reliability (internal consistency)

The Cronbach's alpha statistic was 0.925, which is indicative of excellent internal consistency. Furthermore, there was no evidence of collinearity within the seven items (tolerance 0.213 to 0.429, VIF 2.33 to 4.704).

### Construct validity

*Correlations *between each item of the WRVAS scale and total score and domains of the SF-36v2 are displayed in Table 5. Where significant correlations were found, higher scores on the WRVAS were associated with lower scores on the SF-36v2, which indicates a lower quality of life.

**Table 5:** Correlations between the WRVAS and SF-36v2

	(WR1) Body Curve	(WR2) Rib Prominence	(WR3) Flank Prominence	(WR4) Head Rib Pelvis	(WR5) Head Pelvis	(WR6) Shoulder Height	(WR7) Scapula Rotation	WRVAS Total
PF	-.430**	-.380**	-.338**	-.539**	-.370**	-.432**	-.419**	-.499**
RP	-.350**	NS	NS	-.364**	-.353**	-.361*	-.333**	-.373**
RE	NS	NS	NS	-.267*	NS	NS	NS	NS
BP	-.385**	NS	-.253*	-.388**	-.370**	-.357**	-.297*	-.394**
VT	-.490**	-.322**	-.388**	-.378**	-.313**	-.317**	-.377**	-.445**
MH	-.262*	NS	NS	-.236*	NS	NS	NS	NS
SF	NS	NS	NS	NS	NS	NS	NS	NS
GH	-.438**	-.302*	-.303*	-.391**	-.253*	-.373**	-.336**	-.412**
PCS	-.379**	-.288*	-.267*	-.417**	-.374**	-.395**	-.330**	-.422**
MCS	NS	NS	NS	NS	NS	NS	NS	NS

* *p *< 0.05

** *p *< 0.01

#### Multivariate analysis

The combination of independent variables: total WRVAS score, age and gender, predicted 25.2% of the variance in Physical Component Summary score (*F *= 7.514, *p *< 0.01, *R *= .502, *R*^2 ^= .252). An examination of the independent variables revealed that total WRVAS score and gender contributed to Physical Component Summary scores. Total WRVAS score predicted 17.4% of the variance in Physical Component Summary score (*t *= -3.268, *β *= -.367, *p *= 0.002; *r *= -.417, *r*^2 ^= .174). Gender also predicted 4.7% of the variance Physical Component Summary scores (*t *= 2.311, *β *= .246, *p *= 0.024; *r *= .217, *r*^2 ^= .047).

Sixteen percent of the variance in Mental Component Summary scores was predicted by the combination of total WRVAS score, age and gender (*F *= 4.469, *p *< 0.01, *R *= .401, *R*^2 ^= .160). Of the individual variables, only age and gender predicted Mental Component Summary scores. Age predicted 4.2% of the variance in outcomes on Mental Component Summary scores (*t *= 2.630, *β *= .315, *p *= 0.011; *r *= 206, *r*^2 ^= .042), while gender predicted 5.7% of the variance in outcomes on the dependent variable (*t *= -2.358, *β *= -.266, *p *= 0.021; *r *= -.239, *r*^2 ^= .057).

## Discussion

The aim of the current study was to investigate the psychometric properties of the WRVAS on an adult sample of patients with scoliosis. Floor effects were present for all items of the WRVAS, indicating that respondents may not have been aware of minor scoliosis deformity. The internal consistency was excellent (Cronbach's alpha 0.925) and indicated strong correlations between all items of the WRVAS. However none of the WRVAS items were redundant, as statistical testing revealed no evidence of colinearity. The psychometric properties obtained in the current study including floor and ceiling effects, internal consistency and collinearity were comparable to a previous study by Pineda et al [24] utilizing a predominantly adolescent sample. This suggests that the WRVAS is similarly reliable when administered to adult and adolescent samples.

Analysis of construct validity revealed that patient self assessment of deformity shared a stronger association with physical aspects of quality of life. Items of the WRVAS shared a consistent negative correlation with the Physical Functioning, Vitality and General Health subscales of the SF-36v2, and the Physical Component Summary score. Construct validity outcomes of the current study differ from those of Pineda et al [24], which were that the WRVAS items demonstrated a stronger and more consistent relationship with the Mental Health and Self Image domains of the SRS-22, compared to Pain and Function. One possible explanation is the difference in age group for each study, and normative body image concerns for adolescents compared to older adults. A significant proportion of participants in Pineda et al's [24] study were adolescents, while all participants in the current study were aged over 18 years of age (M = 33 years). The body image literature indicates a preoccupation with appearance during adolescence, with a gradual shift in focus to concerns with health and functionality of the body as individuals age [33]. This assertion is suggested in the adult scoliosis literature, with patients reporting greater limitation in physical aspects of HRQL compared to population norms and control groups more consistently than disruptions in psychological HRQL. Although psychosocial studies demonstrate that appearance is still a valid body image issue for adults with scoliosis, most patients seem to find physical health problems associated with scoliosis more limiting in their daily lives [19].

### Limitations

As previous authors have noted [23,25] the current version of the WRVAS appeared to possess limited face validity in instances where the patient's condition differed from the item depictions of a right thoracic curvature. In the current study, eleven participants made notes referring to their own scoliosis stating how their conditions differed from the illustrations. Of these respondents, two stated that their curvatures were to the left, two reported lumbar curvatures, two reported double major curvatures, three reported that their results were pre or post surgical, and three stated that they had other conditions connected to their scoliosis.

There were two further limitations of the current study that have already been acknowledged. Firstly, there was a small sample size in general, and an unequal distribution of males and females. It is likely that gender is a salient issue in the measurement of body image in adults with scoliosis, given the practically significant results obtained despite the small sample size.

Secondly, scoliosis qualifiers such as curve magnitude and treatment type were not collected from the sample. While this data would have provided more information and enabled for greater statistical control of possible confounding variables, the results obtained still provided useful information with this omission as the purpose of the study was to evaluate patient perceptions of deformity and HRQL. Previous studies have demonstrated a strong correlation between curve magnitude and total WRVAS score in younger participants [23,24,34,35]. However it is unlikely that radiographic indicators would have been useful for the adult sample assessed in the current study, as HRQL demonstrates a poor relationship with radiographic measures in adulthood, with the exception of sagittal balance.

## Conclusion

The results of the current study confirm that the WRVAS is a psychometrically valid tool for use with adult scoliosis populations. These findings add to the complexity of body image data in the scoliosis literature, as it was suggested that physical health factors such as pain and functional capacity are especially salient to body image amongst adult patients. Outcomes of the current study indicate that there is scope for improvement of the WRVAS by increasing the scope of curvature patterns represented, and incorporating items salient to age factors such as kyphosis and lordosis. Furthermore, more comprehensive assessment of HRQL and body image could be achieved by asking patients to evaluate aspects of deformity measured by the WRVAS against body image and HRQL outcomes. Such outcomes include social functioning, satisfaction with appearance, attractiveness, pain, physical functioning and emotional functioning. Measures of these variables could be adapted from previous questionnaires including the SRS, SF-36 or BSSQ – Deformity instruments, all of which have been previously validated on scoliosis populations.

## Authors' contributions

MJT: Data collection and analysis, writing of the manuscript, second and third revisions

NDM: Writing of the manuscript, advice on methodology, assistance in revision of earlier drafts
